# Microalgae—Sustainable Source for Alternative Proteins and Functional Ingredients Promoting Gut and Liver Health

**DOI:** 10.1002/gch2.202200177

**Published:** 2023-04-25

**Authors:** Yahav Eilam, Hamdan Khattib, Noam Pintel, Dorit Avni

**Affiliations:** ^1^ Sphingolipids, Active Metabolites, and Immune Modulation Laboratory MIGAL – Galilee Research Institute Tarshish 2 Kiryat Shemona North 1101600 Israel; ^2^ Department of Biotechnology Tel Hai College Upper Galilee North 1220800 Israel

**Keywords:** alternative proteins, bioactive compounds, inflammatory bowel disease, microalgae, microbiome, non‐alcoholic fatty liver disease, peptides

## Abstract

Dietary proteins derived from animal sources, although containing well‐balanced profiles of essential amino acids, have considerable environmental and adverse health effects associated with the intake of some animal protein‐based products. Consuming foods based on animal proteins carries a higher risk of developing non‐communicable diseases such as cancer, heart disease, non‐alcoholic fatty liver disease (NAFLD), and inflammatory bowel disease (IBD). Moreover, dietary protein consumption is increasing due to population growth, posing a supply challenge. There is, therefore, growing interest in discovering novel alternative protein sources. In this context, microalgae have been recognized as strategic crops that can provide a sustainable source of protein. Compared to conventional high‐protein crops, using microalgal biomass for protein production presents several advantages in food and feed in terms of productivity, sustainability, and nutritional value. Moreover, microalgae positively impact the environment by not exploiting land or causing water pollution. Many studies have revealed the potential of microalgae as an alternative protein source with the added value of positive effects on human health due to their anti‐inflammatory, antioxidant, and anti‐cancer properties. The main emphasis of this review is on the potential health‐promoting applications of microalgae‐based proteins, peptides, and bioactive substances for IBD and NAFLD.

## Graphical abstract

The effect of microalgae‐based bioactive compounds on gut and liver health. Microalgae‐based bioactive compounds, including protein, peptides, pigments, lipids, and polyphenols, have a positive impact on the inflammatory process and related diseases, inflammatory bowel disease, and non‐alcoholic fatty liver disease, and can potentially inhibit their progression.

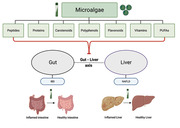

## 1 Introduction

Our global food system contributes primarily to biodiversity loss, environmental pollution, and overexploitation of terrestrial and aquatic sources. It is the third and most significant contributor to climate change after the energy industry^[^ ^1^ ^]^ and population growth (reaching an expected 9.7 billion people by 2050).^[^ ^2^ ^]^ The potential destruction of ecosystems and habitats could hamper the sustenance of human populations. Thus, there is an urgent need to reform food systems through changes in global dietary patterns, and by protecting and partitioning land for nature and farming in a way that will support biodiversity. Moreover, the COVID‐19 pandemic and the current war in Europe, which are affecting food security worldwide, emphasize the need to find alternative solutions for the food industry.

Due to population growth, the development of the middle classes, and society's awareness of increasing protein consumption, the demand for dietary proteins is on the rise.^[^ ^3^ ^]^ In response to this rapid change in the food market, aquaculture and the food industries have been working closely for the last few years to increase the output of protein products in terms of both growth and production.^[^ ^4^ ^]^ Proteins derived from animals are considered of higher quality in terms of their amino acid profile, which is usually superior to those of most alternative protein sources. However, the search for protein alternatives is driven by the negative health effects associated with the dietary consumption of some animal‐based products, red meat in particular.^[^ ^5^, ^6^ ^]^ In the last decade, researchers and food product development experts have been working diligently and collaboratively to solve this problem. In this context, microalgae have proven to be a strategic source of protein due to their sustainability, enormous biological diversity, and high protein content.^[^ ^6^ ^]^ Microalgae enable the high production of proteins through many different manipulations and technologies; although they may not be able to completely replace the consumption of animal‐based proteins, they may help reduce it.^[^ ^7^ ^]^

Advances have been made in reducing the consumption and production of animal foods, but the ongoing waste associated with feeding animals through crops remains.^[^ ^8^ ^]^ Animal feed continues to represent a massive net loss of available global proteins and calories.^[^ ^9^ ^]^ Algae and microalgae, in particular, grow ten times faster than traditional crops, such as wheat, corn, and hay, and may provide high amounts of protein, amino acids, and other micronutrients, such as fatty acids, and in particular omega‐3 fatty acids, vitamins, minerals, and antioxidants.^[^ ^10^ ^]^ Unlike agriculture, aquaculture used for the production and cultivation of microalgae does not require large terrestrial areas.^[^ ^11^ ^]^ In addition, it does not result in the pollution of drinking water or marine ecosystems through the use of fertilizers, for example.^[^ ^4^ ^]^

The last few decades have seen dramatic technological, cultural, and social advances in food production and consumption.^[^ ^12^ ^]^ Yet, many people are in poor health due to western, high‐fat, high‐carbohydrate diets, and low mobility. This poor nutrition and lifestyle cause nutrient deficiencies, obesity, impaired brain development, a weak immune system, increased infections, and chronic inflammatory disease.^[^ ^13^ ^]^ Consumption of animal‐based products exposes humans to excess saturated fat, cholesterol, estrogens, and pathogenic microorganisms, while these diets are lacking in fiber, complex carbohydrates, antioxidants, and other components needed for well‐being.^[^ ^14^ ^]^ Consumption of animal‐based products increases risks for non‐communicable diseases such as cardiovascular disease,^[^ ^15^ ^]^ cancer,^[^ ^16^ ^]^ diabetes,^[^ ^17^ ^]^ non‐alcoholic fatty liver disease (NAFLD),^[^ ^18^ ^]^ inflammatory bowel diseases (IBD),^[^ ^19^ ^]^ and other chronic illnesses.^[^ ^20^ ^]^ These pathologies are characterized by inflammatory status on the one hand, and alteration of the gut microbiota on the other. Whereas animal‐based diets are consistently associated with a lower diversity of gut bacteria and irritation of the gut barrier, plant‐based diets are linked to an abundant richness of bacteria that reduce the risk of inflammation.^[^ ^21^ ^]^ Nevertheless, low‐ to mid‐income populations are tempted by the low prices and availability of animal‐based products. In contrast, alternative protein products of high quality are expensive and hard to find or identify. Nevertheless, the global crises surrounding food security and environmental issues require transformative solutions. The demand for protein is growing, but livestock‐based protein production is becoming less sustainable; therefore, alternative sustainable protein sources must be found, including cultured meat, plant‐based protein, insect protein, fungi, and microalgae.^[^ ^14^ ^]^

Microalgae are viable alternatives to animal‐based proteins because they are high in protein and essential amino acids.^[^ ^22^ ^]^ Microalgae generally have very high protein contents—up to 70%, significantly higher than the traditional plant protein sources (soy: 35% and chickpea: 18%) and comparable to animal protein sources (turkey: 63% and beef: 50%).^[^ ^23^ ^]^

The fact that microalgae are frequently recognized as environmentally beneficial is another element that makes them attractive.^[^ ^7^ ^]^

Despite the large variety of algal protein sources, functional peptides from microalgal protein hydrolysates have only scarcely been described.^[^ ^24^ ^]^ The microalga's biomass consists of over 50% protein, used mainly as animal feed.^[^ ^25^ ^]^ However, enzymatic hydrolysis can convert microalgal proteins into value‐added food products with enhanced functional properties. Thus, there is a growing interest in producing effective bioactive peptides and other bioactive compounds from marine microalgae.^[^ ^26^ ^]^ A comprehensive understanding of the quality and functional properties of proteins found in microalgal hydrolysates would help elucidate their potential as additives and ingredients for food and dietary products.^[^ ^27^ ^]^

This review focuses on the use of microalga‐based proteins, peptides, and other active compounds as functional foods to promote gut and liver health. In an effort to advance the research that will result in product development and commercialization of microalgae to satisfy the increasing demand for sustainable alternative protein products, the review will present the current challenges, potential barriers, and impacts on environmental factors and new markets.

## 2 Microalgae

Microalgae are a vast group of mainly autotrophic unicellular prokaryotic and eukaryotic organisms. Despite the estimated 200,000 microalgal species (according to AlgaeBase (www.algaebase.org)^[^ ^28^ ^]^ and the increasing descriptions of new species in recent years, only a few species have been investigated extensively; these include *Dunaliella*,^[^ ^29^ ^]^ *Chlorella*,^[^ ^30^ ^]^ *Isochrysis*,^[^ ^31^ ^]^ *Nannochloropsis*,^[^ ^32^ ^]^ *Haematococcus*,^[^ ^33^ ^]^ and *Spirulina*.^[^ ^34^ ^]^

Among these 200 000 species, a few of them have been consumed since ancient times, and are classified as GRAS (generally recognized as safe) food sources. These are *Spirulina, Chlorella*, *Dunaliella*, *Haematococcus*, *Schizochytrium*, *Porphyridium cruentum*, and *Crypthecodinium cohnii*. In addition, products derived from microalgae and classified by the United States Food and Drug Administration (FDA) as GRAS include oils obtained from *Schizochytrium* and *Ulkenia*, whole microalgal protein powder, and a lipid ingredient derived from *Chlorella* (see **Table** **1**). Several microalgal products have been approved in the European Union as novel food ingredients, such as *Tetraselmis chuii* (approved in 2014).

**Table 1:** Microalgal life‐cycle assessments and composition

Biomass type	[€ kg^−1^]	Nutritional quality (PDCAAS* %)	Safety	Product Carbon Footprint (PCF)***	Proteins (% w w^−1^)	Carbohydrates (% w w^−1^)	Lipids (% w w^−1^)	References
*Arthrospira platensis* (Spirulina)	9.8–39.2 kg^−1^	48%	Approved 1997	7.88–12	53.6–62.9	5.7–10.2	12–11	[101, 102, 103, 104, 105, 106]
*Chlorella vulgaris Pyrenoidosa luteoviris*	19.6–39.2 kg^−1^	Known for some strains—*C. vulgaris*—0.64–64%^2^	Approved 1997	Unknown	51–58	12–17	14–22	[100, 103, 105, 107, 108]
*Tetraselmis chuii*	Unknown	50–60%	Approved 2014	Unknown	35–40	30–32	5–8	[64, 103, 105, 109]
*Nannochloropsis oceanica*	Unknown	35.5%	Pending (Approval)	Unknown	22–37	28–40	15–21	[103, 105, 110, 111]
*Schizochytrium* sp.	Unknown	Unknown	*Schizochytrium* based‐oil approved 2020	Unknown	12	38–71	32	[68, 105, 111]
*Haematococcus pluvialis*	Unknown	Unknown	Astaxanthin‐rich oleoresin from *H. pluvialis* algae approved by EFSA (2017) and reaffirms to be limited to up 8mg per day (2020)	Unknown	48	27	15	[105, 112]

Microalgae are recognized as a diverse source of bioactive molecules with a physiological role in their environment; they can modulate their metabolism in response to environmental conditions. Thus, microalgae are a potential natural source of bioactive compounds such as polysaccharides, lipids, proteins, peptides, carotenoids, vitamins, pigments, phenolic compounds, and fatty acids. These compounds have potential health applications due to antioxidant, antimicrobial, anti‐inflammatory, anticancer, antidiabetic, antihypertensive, antihyperlipidemic, and antiobesity effects.^[^ ^35^, ^36^ ^]^ Because microalgae produce various primary and secondary metabolites, they can serve as therapeutic agents for many health disorders and are used in the cosmetic, food, and pharmaceutical industries.^[^ ^37^, ^38^, ^39^ ^]^ Bioactive compounds isolated from microalgae have been shown to have anti‐inflammatory properties that can be used to treat a variety of inflammatory diseases, through inhibition of pro‐inflammatory cytokine production and reduced expression of inflammatory genes.^[^ ^40^ ^]^ For example, the highly nutritious blue‐green *Spirulina* microalgae (*Arthrospira platensis*) is used as a nutraceutical food supplement worldwide. It also has therapeutic properties, including anti‐inflammatory activity.^[^ ^41^ ^]^ It contains the pigment‐binding protein phycocyanin, which inhibits the formation of pro‐inflammatory cytokines such as tumor necrosis factor alpha (TNF‐*α*), reduces the production of prostaglandin E2, and inhibits the expression of cyclooxygenase‐2 (COX‐2).^[^ ^42^ ^]^

### 2.1 Microalga‐Based Proteins and Peptides

Microalgal biomass protein content varies from 30 to 80 mass percent: for example, *Chlorella vulgaris* contains 51–58% protein; *A. platensis* (*Spirulina*) has 60–71%; *T. chuii* has 31–46%*; Nannochloropsis oceanica* has 35–44%; *Dunaliella salina* has 50–80%; and *Galdieria sulphuraria* has 62%.^[^ ^43^, ^44^ ^]^ Microalgae produce 2.5 to 7.5 tons of protein per hectare each year. Some microalgae, such as *Spirulina* and *Chlorella*, are considered a good source of protein with favorable amino acid composition and are being marketed as such.^[^ ^44^ ^]^ The human body cannot synthesize essential amino acids and must obtain them from an outside source, usually food. Several different important amino acids and proteins, which can be used in food and protect against diseases, are produced in greater amounts by many different species of microalgae. Algae‐based proteins are rich in essential amino acids such as leucine, arginine, and lysine that make up 7% of all proteins; other essential amino acids, such as isoleucine, phenylalanine, threonine, and valine, typically make up 4% of all proteins.^[^ ^45^ ^]^

Algae‐based proteins as phycocyanin, peptides as Leu‐Asn‐Gly‐Asp‐Val‐Trp (*Chlorella ellipsoidea*), and essential amino acids such as arginine, histidine, isoleucine, leucine, lysine, and methionine provide potent health benefits or are essential for cells and tissues to carry out their functions.^[^ ^46^, ^47^ ^]^

Interest in microalgae‐derived bioactive peptides has increased, due to their numerous beneficial health effects.^[^ ^36^ ^]^

Today, half of the global protein and peptide market is sourced from terrestrial plants; by 2054, it is expected that these could be replaced by microalga‐based proteins.^[^ ^48^ ^]^ Microalgal proteins and peptides have been exploited as part of the ongoing trend to seek novel food ingredients and alternative proteins with health benefits. Suttisuwan et al, demonstrated a reduction in pro‐inflammatory cytokine gene expression by a microalga‐based peptide fraction and presented a potentially rich source of microalgal peptides for the development of new functional food ingredients and medicinal compounds against inflammation.^[^ ^49^ ^]^

Various biological effects of *Chlorella*‐11 peptide from the green algae *Chlorella pyrenoidosa* have been demonstrated. This peptide—Val‐Glu‐Cys‐Tyr‐Gly‐Pro‐Asn‐Arg‐Pro‐Gln‐Phe—decreased bacterial lipopolysaccharide (LPS)‐induced production of the pro‐inflammatory cytokine monocyte chemoattractant protein‐1 in RAW264.7 macrophages. In addition, it inhibited the production of intercellular cell adhesion molecule‐1, endothelial cell selection, and vascular cell adhesion molecule‐1 in endothelial cells,^[^ ^50^ ^]^ as well as nitric oxide production in a dose‐ and time‐dependent manner,^[^ ^51^ ^]^ and the expression and activity of nuclear factor‐kappa B (NF‐ƙB).^[^ ^52^ ^]^ Thanh‐Sang et al.,^[^ ^26^ ^]^ purified two peptides—LDAVNR and MMLDF—from an enzymatic hydrolysate of *Spirulina maxima*; these peptides significantly suppressed cytokine generation by endothelial cells, indicating their potent anti‐inflammatory benefits. In the last decade, in vitro and in vivo studies have shown the health benefits and biological activities of bioactive peptides obtained from *Spirulina platensis*, including antioxidant, anticancer, antidiabetes, and antiobesity activity.^[^ ^53^ ^]^ Ko et al.,^[^ ^54^ ^]^ isolated a peptide from *C. ellipsoidea* (Leu‐Asn‐Gly‐Asp‐Val‐Trp) with peroxyl radical, 1,1‐diphenyl‐2‐picrylhydrazyl‐ and hydroxyl radical‐scavenging activity. Two peptides isolated from *Nannochloropsis oculata* (Gly‐Met‐Asn‐Asn‐Leu‐Thr‐Pro and Leu‐Glu‐Gln) demonstrated antihypertensive properties through their inhibition of angiotensin‐converting enzyme activity.^[^ ^55^ ^]^ This illustrates the great potential of using microalgae‐based peptides to improve health.

Phycocyanin isolated from *Spirulina* and many other microalgal species is a biliprotein with antioxidant and anti‐inflammatory activities.^[^ ^56^ ^]^ It was evaluated in a mouse model, where in addition to its inhibitory effect on IgE activity, it reduced vascular permeability and consequently, inflammation.^[^ ^57^ ^]^

Due to the tremendous commercial potential of microalgal proteins and peptides, establishing an economic and mass‐produced method for their extraction might be complex but is essential for reducing the costs of drugs and other applications as novel functional ingredients and alternative proteins for the food industry.

#### 2.1.1 Protein Production

**Figure 1:**
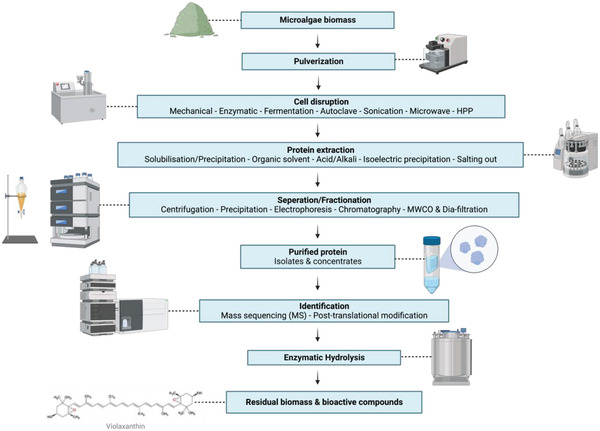
Production of microalgal proteins and bioactive compounds (created with BioRender.com).

Conventional protein‐extraction techniques generally produce low yields because the proteins are degraded by the process’ extreme temperature and pH conditions. Thus, there is a need for novel, non‐thermal extraction methods that employ enzymes and “eco‐friendly” procedures to increase extraction efficiency and decrease negative impacts on the environment. In the currently used methods, proteins are extracted with sugars, polyphenols, and other compounds. Thus, isolation and purification procedures are necessary. The residual biomass, produced as a result of the extraction procedure, contains essential bioactive compounds that could be used for food applications and could be considered for whole‐biomass usage. **Figure** **1** schematizes the protein‐extraction procedure, and the production of bioactive peptides and other bio‐compounds.

Peptide extraction generally involves two or more purification methods to separate the biologically active peptides in the hydrolysate: selective precipitation, chromatographic ultrafiltration, and ultrafiltration.^[^ ^58^ ^]^ The purification methods that are currently being used by the industry to extract marine microalgal peptides are complicated and difficult to apply on a large scale.^[^ ^59^ ^]^ When the microalgae are cultured under non‐stressful conditions, they produce large amounts of protein;^[^ ^60^ ^]^ however, under stress conditions, such as high salinity or nitrogen starvation, they accumulate excess amounts of lipids and carbohydrates.^[^ ^61^ ^]^

In evaluating the suitability of microalgal proteins for human consumption, total amino acid, essential amino acid contents, protein digestibility, bioaccessibility, and bioavailability must be considered. The protein digestibility corrected amino acid score (PDCAAS) values for meat and whey proteins are determined as 1.^[^ ^62^ ^]^ Plant and microalgae‐based proteins are usually lower than 1.^[^ ^62^, ^63^ ^]^ This might be due to the structure of the algal cell wall: Its components may interact with the microalgal proteins when digested, or the proteins may be retained in the cell wall, limiting full digestion and functioning.^[^ ^63^ ^]^ This issue of digestibility must be taken into consideration when evaluating the quality of microalgal amino acids, essential amino acids, and total protein.^[^ ^64^ ^]^ Different in vivo and in vitro methods have been tested to evaluate protein quality. The most common is the in vitro protein digestibility method, which uses enzyme degradation of the tested protein to simulate the gastric and pancreatic phases and compares the level of digestibility to a fully digestible reference protein.^[^ ^43^ ^]^ Breaking down the cell wall can increase the percentage of digestible proteins.^[^ ^7^ ^]^ However, the cell wall protects these proteins against pH changes and maintains them in a nurturing environment, indicating that whole‐cell algal proteins that remain in their natural environment are most functional.^[^ ^65^ ^]^ The Food and Agriculture Organization (FAO) recommends the use of in vivo techniques for a precise assessment of protein quality because the biological efficacy of the protein can only be thoroughly determined in humans or animals.^[^ ^45^ ^]^

Notably, microalgae‐based proteins when compared to other plant‐based proteins such as beans, peas, and chickpeas have higher PDCAAS value.^[^ ^66^ ^]^

Proteins also contribute to microalgal rheology and stability during production and storage and are a source of bioactive peptides with a wide range of health effects upon consumption.^[^ ^67^ ^]^ Moreover, numerous microalgal species produce enzymes of a commercial interest with a broad range of potential uses, such as the antioxidants superoxide dismutase and catalase, and enzymes with peroxidase activity. Microalgal proteins are generally highly soluble at high pH and show minimal solubility at pH less than 4. The amphiphilic nature of the proteins confers excellent emulsification ability.^[^ ^68^ ^]^

#### 2.1.2 Separation of Microalgal Proteins

Depending on the technique used to disrupt the microalgal cells, the medium will contain proteins, fine particles, intact cells, disrupted or damaged cells, and other undesired compounds and by‐products such as nucleic acids and ribosomes.^[^ ^69^ ^]^ Separation of microalgal proteins from the cellular debris depends mainly on their dispersibility in water, where the protein‐rich aqueous phase is separated from the solid phase,^[^ ^70^, ^71^ ^]^ usually by centrifugation.^[^ ^72^, ^73^ ^]^ However, the parameters required for this technique—high acceleration (e.g., 10 000× *g*) and low temperatures—might limit its scale‐up to microalgal farms.^[^ ^72^, ^73^ ^]^ Moreover, separation by centrifugation generates a protein‐rich supernatant with high chlorophyll content, because this nonpolar pigment adheres to the fine particles present in the aqueous phase, probably as a colloidal dispersion.^[^ ^71^, ^72^ ^]^ An alternative technique to separate out the protein‐rich liquid phase is filtration or ultrafiltration.^[^ ^74^ ^]^

The most critical parameter for the efficient dispersion of proteins is ionic strength,^[^ ^73^ ^]^ and therefore pH adjustment presents a simple and low‐cost means of improving protein dispersion.^[^ ^70^, ^74^ ^]^ Most commercial microalgal proteins have a specific range of isoelectric points, for example, 5–7 for *Haematococcus pluvialis* and 4–5.5 (major group) and 6–8 (minor group) for *C. vulgaris*.^[^ ^74^ ^]^ In contrast, the isoelectric point of *A. platensis* ranges from 2.8 to 3.5.^[^ ^75^ ^]^ Thus, most microalgal proteins can be dispersed by increasing the solution pH. Protein recovery yields can be further enhanced by combining the pH adjustment with the mechanical technique of cell disruption^[^ ^74^ ^]^ and temperature.^[^ ^73^ ^]^ However, the use of harsh conditions for protein extraction should be considered with caution. High‐pressure extraction of *C. vulgaris* protein at alkaline pH was shown to affect protein folding, resulting in its aggregation and loss of emulsification properties.^[^ ^74^ ^]^

More recently, a three‐phase partitioning technique has been proposed for microalgal protein separation, whereby the microalgal components are fractionated into nonpolar (upper) and polar (lower) phases, and the proteins are held in the middle phase through a combination of ammonium sulfate for protein precipitation, and butanol to increase protein buoyancy. Optimized conditions for three‐phase partitioning resulted in a *Chlorella pyrenoidosa* extract with a protein concentration of 78% (wt/wt).^[^ ^76^ ^]^ However, in that study, a high concentration of ammonium sulfate (20–50%, wt/wt) and solvents was required to achieve the high yields. To adopt the three‐phase partitioning technique for the commercial production of microalgal proteins, the input of chemicals must be reduced.

Use of a high‐pressure homogenizer to disrupt microalgal cells involves pumping the harvested biomass through a valve that collides with an impact ring. The high pressure of the fluid that is accelerated along the stationary surface of the valve (shear force) and the cavitation resulting from the pressure drop induced by the shear stress are what disrupt the cells. However, the amount of applied pressure is highly dependent on the microalgal species, the biomass concentration, and the growth conditions under which the strain was cultivated. The main disadvantages of this technique are the challenges involved in controlling the temperature production of particulates and the release of various bio‐compounds; this increases the cost of isolating products of interest.^[^ ^77^, ^78^ ^]^

#### 2.1.3 Concentration of Microalgal Proteins

Microalgal protein concentration is affected by the culture conditions and the species. In autotrophic organisms, cell concentration, and hence biomass productivity, are restricted by the phenomenon of shadowing, which limits the cells’ exposure to sunlight. Some of these species can be cultivated heterotrophically in fermenters, conferring some advantages over autotrophic culturing, such as the possibility of larger scale growth, compliance with FDA‐approved standards and protocols for industrial fermenters, and the ability to reach higher cell densities because cell shadowing is no longer an issue. As a result, microalgae grown heterotrophically have higher protein concentrations than those grown autotrophically, likely due to the reduction in photosynthetic pigments, mainly nitrogen‐rich chlorophyll a, which shifts the metabolism to protein production.^[^ ^79^ ^]^

Several methods are available for concentrating the proteins, depending on their characteristics. Proteins are often precipitated using trichloroacetic acid or ammonium sulfate. However, these chemicals remain in the protein concentrate, and their removal might be required for food and feed applications, among others.^[^ ^73^, ^80^ ^]^

Ultrafiltration does not require the addition of salts or acids, making it suitable for concentrating proteins. It makes use of semipermeable membranes through which small molecules (e.g., water and salts) can flow, while larger molecules, such as proteins, are retained.^[^ ^73^ ^]^ Another chemical‐free technique that is primarily employed in protein concentration is freeze‐drying, whereby water is removed by sublimation at low temperature and pressure.^[^ ^73^, ^74^ ^]^ The reduction in residual water content enhances protein stability, enabling the storage of freeze‐dried proteins at room temperature, with minimal loss of their activity. However, the salt content of the microalgal protein extract might render freeze‐drying unsuitable, because this technique increases the concentration of salt in the protein extract after water removal, which may change the ionic strength and pH, resulting in protein denaturation.^[^ ^81^ ^]^

#### 2.1.4 Hydrolysates

Because the lower‐value products, such as protein concentrates and whole‐cell protein, are relatively expensive to produce, they cannot compete with the prices of these products from traditional sources (3–5 USD kg^−1^).^[^ ^58^ ^]^ One way to improve the biomass value is protein hydrolysis,^[^ ^82^ ^]^ because the final product has high solubility, digestibility, and bioactivity, increasing its market value. The use of lipid‐extracted algae and enzymatic protein hydrolysates to increase protein solubility by breaking the denatured protein down into smaller fragments have been suggested to provide an excellent source of protein for human nutrition, with better gastrointestinal absorption than for proteins and free amino acids.^[^ ^83^ ^]^ Currently, most protein hydrolysates are derived from soybean and whey. Soybean hydrolysates are not the most sustainable due to the growing demand for crop areas,^[^ ^84^ ^]^ and the unfavorable impact of agriculture on soil fertility levels, contamination of water sources with agrochemicals, and contribution to habitat loss and soil degradation. Dairy whey proteins also face a sustainability issue; dairy wastewater has a high concentration of organic elements, such as nitrates, phosphorus, proteins, lipids, and carbohydrates, which raises the chemical and biological oxygen demand.^[^ ^85^ ^]^ Dairy pollutants are released into water bodies, which may accelerate their degradation but also results in a strong odor. Dairy wastewater also ends up contaminating natural water bodies. The nutritionally dense wastewater promotes the rapid growth of bacteria, which can capture a significant quantity of oxygen in the aquatic environment. As a result, both animals and plants become anaerobic, which ultimately contributes to mass extinction.^[^ ^86^ ^]^ Therefore, the use of protein sources from microalgae may help improve the sustainability of the food industry. Protein hydrolysates have been integrated into a number of formulations to improve nutritional and functional properties.^[^ ^87^ ^]^

#### 2.1.5 Digestibility

Investigation of the bioavailability and digestibility of novel protein sources is essential. Factors that can influence a protein's digestibility include its conformation, anti‐nutritional factors, and the downstream process used for its extraction. Proposed approaches to simplifying the evaluation of protein quality are based on the contents and bioavailability of essential amino acids.^[^ ^88^ ^]^ For example, the PDCAAS is the ratio of the limiting essential amino acid (i.e., the amino acid that differs most in content from the reference) in the protein and its amount in the FAO reference. This ratio is then multiplied by the digestibility of the protein.^[^ ^88^, ^89^ ^]^

As already noted, animal‐based proteins (of meat and dairy origin) have PDCAAS values of 1 and are therefore considered complete protein sources, because of both their amino acid content and their digestibility values. Most of the PDCAAS values found for different species of microalgae are lower than 1, due to the components found in the walls of the microalgal cells that bind to the available algal proteins, preventing their digestion.^[^ ^68^ ^]^ Because the cell wall makes microalgal whole‐cell proteins difficult to digest, the peptides obtained by enzymatic hydrolysis are favored by researchers. Moreover, these enzymatic hydrolysates are highly digestible, and a sustainable and reliable source that can serve to replace protein sources such as soy, whey, and fish protein in nutritional supplements.^[^ ^90^ ^]^ Cyanobacteria were found to be most easily digested, especially *A. platensis* F&E‐C256 (78% dry matter, 86% organic matter, 79% carbohydrate, and 82% crude protein digestibility), *Chlorella sorokiniana* F&E‐M‐M49 and *C. vulgaris* sold by Allma. Marine species, such as *Tetraselmis suecica*, *Phaeodactylum tricornutum*, *Nannochloropsis* sp., and *Porphyridium purpureum*, were the least digestible.^[^ ^91^ ^]^ The difference is probably due to their cell wall structures. *Nannochloropsis*’ thick cell wall, made up of cellulose and algaenans, might reduce digestibility.^[^ ^63^ ^]^ *Porphyridium* cells are covered by polysaccharides that can form stable complexes, and proteins can facilitate the cell's access to proteolytic enzymes. In contrast, the cell wall of green algae such as *T. suecica* is composed of cellulose, hemicellulose, pectic compounds, and glycoproteins; all of these compounds can interfere with the activity of digestive enzymes. Taken together, cell lysis is vital to increase the digestibility of microalgae.

There are other methods of cellular disruption based on the application of electrical fields, such as pulsed electric field (PEF) and moderate electric field (MEF) technologies. PEF is considered a non‐thermal technology; it works by means of high‐intensity electrical pulses on a sample. This technology is based on the electroporation of the wall of microalgal cells, causing an increase in cell permeability.^[^ ^92^ ^]^ This permeability allows easy access for certain solvents in the extraction process.^[^ ^93^ ^]^ Using this technology on *C. vulgaris* and *Nannochloropsis salina*, there was some evidence of protein leakage after treatment.^[^ ^94^, ^95^ ^]^ Using this technology with *C. vulgaris* achieved a total protein extraction of 96%.^[^ ^96^ ^]^ MEF is a non‐pulsing approach characterized by the presence of an alternating current without treatment time restrictions.^[^ ^92^ ^]^ MEF must be applied at high frequencies because the use of low frequencies may lead to the release of other metal ions that may react with nutrients in the sample and form radicals.^[^ ^97^ ^]^ The optimal electrical frequency range is 50 Hz to 25 kHz.^[^ ^98^ ^]^ Moreover, the use of low frequencies can contribute to the ventricularization of biological membranes as observed in some bacteria.^[^ ^99^ ^]^ The effects of this method on the cell structures of microalgae have not yet been clarified.

Interestingly, using mechanical cell wall disruption techniques not only produce more protein but also affects the PDCAAS value. Wang et al., (2020) found the PDCAAS of the disrupted biomass of *C. vulgaris*, *Chlorella sorokiniana*, and *Acutodesmus obliquus* increased to 0.77, 0.81, and 0.46 compare to the raw biomass (PDCAAS values of 0.63, 0.64, and 0.29, respectively).^[^ ^100^ ^]^

### 2.2 Other Microalgae‐Based Bioactive Compounds

A variety of compounds produced by microalgae are valuable to the food, nutraceutical, cosmetic and pharmaceutical industries.^[^ ^113^ ^]^ They include proteins, essential amino acids, polysaccharides, lipids, vitamins, pigments, and other active components^[^ ^114^ ^]^ with health‐promoting functions.^[^ ^115^ ^]^ Microalgal species of *Chlorella*, *Scenedesmus*, *Arthrospira*, *Spirulina*, *Nostoc*, and *Aphanizomenon* are well‐known for their production of active metabolites that hold promise for nutraceuticals and pharmaceuticals (**Table** **2**).^[^ ^113^, ^116^ ^]^ For example, the ketocarotenoid astaxanthin is produced mainly by *H. pluvialis*, *Chlorella zofigiensis* and *Chlorococcum* sp.^[^ ^117^ ^]^ However, it is found in the highest amounts in the green algae *H. pluvialis*, making up 4–5% of the algae's dry weight. Its potent antioxidative, anticancer, antidiabetic, anti‐inflammatory activities, gastroenterological, and hepatic effects confer its many applications in medicine. *β*‐carotene carotenoids have antioxidant potential, with structural and physicochemical properties that may be responsible for their skin‐protective effects against solar radiation damage.^[^ ^118^ ^]^

**Table 2:** Microalgae‐based bioactive compounds and their health potential

Bioactive compounds	Microalgae	Potential activities and mechanisms of action	Effect on IBD/NAFLD	Chemical structure	References
Chlorella‐11: Val‐Glu‐Cys‐Tyr‐Gly‐Pro‐Asn‐Arg‐Pro‐Gln‐Phe (peptide)	*C. vulgaris*	Anti‐inflammatory; decreased LPS‐induced production of the pro‐inflammatory monocyte chemoattractant protein‐1(MCP‐1) in RAW264.7 macrophages	N/A	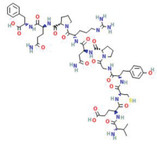	[50]
Leu‐Asn‐Gly‐Asp‐Val‐Trp (peptide)	*Chlorella ellipsoidea*	Antioxidant activity; peroxyl radical, 1,1‐diphenyl‐2‐picrylhydrazyl‐ and hydroxyl radical‐scavenging activity	N/A	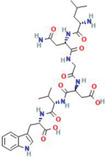	[54]
Gly‐Met‐Asn‐Asn‐Leu‐Thr‐Pro (peptide)	*Nannochloropsis oculata*	Antihypertensive properties through inhibition of angiotensin‐converting enzyme activity	N/A	N/A	[54]
Leu‐Glu‐Glu (peptide)	*N. oculata*	Antihypertensive properties through inhibition of angiotensin‐converting enzyme activity	N/A	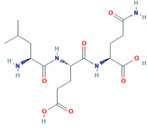	
LDAVNR (peptide)	*Spirulina maxima*	Antioxidant activity; significantly suppressed cytokine generation by endothelial cells	N/A	N/A	[54]
MMLDR (peptide)	*Spirulina maxima*	Antioxidant activity; significantly suppressed cytokine generation by endothelial cells	N/A	N/A	[54]
Sulfated polysaccharides	*Porphyridium*, *Rhodiola reticulata*	Antioxidant	Reduces liver weight and significantly lowers hepatic HMG‐CoA reductase (HMGCR) mRNA protein expression	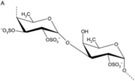	[128]
ß‐1,3‐Glucan	*Chlorella*, *Skeletonema*, *Porphyridium*	Free radical collector, immune system booster, anti‐inflammatory	N/A	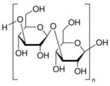	[129]
Astaxanthin	*H. pluvialis*	Antioxidant	Suppresses dextran sulfate sodium (DSS)‐induced histological inflammatory changes, reduces plasma levels of malondialdehyde and 8‐hydroxy‐2‐deoxyguanosine, suppresses mucosal mRNA expression of interleukin (IL)‐1*β*, IL‐6, TNF‐*α*, IL‐36*α*, and IL‐36*γ* and blocks DSS‐induced translocation of NF‐*κ*B, p65, and AP‐1 (c‐Jun) into the nucleus of mucosal epithelial cells	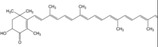	[130]
Phycocyanobilin, phycoerythrobilin	*Spirulina, Porphyridium*	Antioxidant	Resists oxidation by scavenging free radicals, inhibiting the activity of nicotinamide adenine dinucleotide phosphate (NADPH) oxidase, and delaying the activity of antioxidant enzymes. Phycocyanobilin can also be used as an excellent anti‐inflammatory agent to reduce the pro‐inflammatory factors IL‐6 and interferon (IFN)‐*γ* and to upregulate the production of anti‐inflammatory cytokine IL‐10 by inhibiting the inflammatory signaling pathways of NF‐*κ*B and mitogen‐activated protein kinase (MAPK)	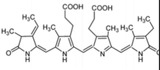	[130]
*β*‐Cryptoxanthin, *β*‐carotenes	*Dunaliella salina*	Anti‐inflammatory, promotes hyaluronan synthesis, antioxidant	N/A	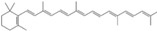	[130]
Ergosterol	*C. vulgaris*	Reduces LPS‐induced inflammatory response	Lowers levels of intracellular triglyceride content in fatty acid‐induced HepG2 cells	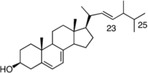	[131]
Eicosatetraenoic acid	*Phaeodactylum*	Anti‐inflammatory	Significantly ameliorates lipid accumulation by suppressing lipid biosynthesis and promoting fatty acid transport and *β*‐oxidation	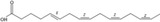	[127]
Phycocyanin	*Arthrospira*	Anti‐inflammatory, antioxidant	Upregulates the expression of AMP‐activated protein kinase (AMPK) phosphorylation and downregulates sterol regulatory element‐binding protein (SREBP)‐1c, upregulates the expression of transcription factor peroxisome proliferator‐activated receptor alpha (PPAR*α*), regulated by AMPK, and its target genes, improves liver inflammatory infiltration by upregulating the expression of PPAR*γ* and downregulates the expression of CD36, IL‐6, and TNF‐*α*	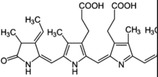	[128]
Lutein	*H. pluvialis*	Anti‐inflammatory	Lowers total cholesterollevels in the serum and total cholesterol and tryglicerids levels in the liver, reduces levels of lipid accumulation in the liver and the content of glutamic pyruvate transaminases, improves insulin sensitivity, increases the expression of key factors in insulin signaling in the liver, such as insulin receptor substrate‐2, phosphatidylinositol 3‐kinase, and glucose transporter‐2 at the gene and protein levels, increases expression of peroxisome‐activated receptor‐*α* and sirtuin 1, which are associated with lipid metabolism and insulin signaling	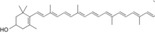	[132]
Eicosapentaenoic acid (EPA)	*Tetraselmis* sp.	Anti‐inflammatory	Enhances the conversion of EPA to 18R oxidized products, including RvE1, which carry strong anti‐inflammatory signals, reducing serum anti‐2,4,6‐trinitrobenzene sulfonic acid IgG, decreasing leukocyte infiltration and pro‐inflammatory gene expression, including IL‐12 p40, TNF‐*α*, and inducible nitric oxide synthase. Leukocyte‐mediated anti‐tissue regulation and pro‐inflammatory gene expression	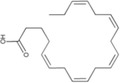	[133]
Docosahexaenic acid (DHA)	*Tetraselmis* sp.	Anti‐inflammatory	Significantly improves insulin sensitivity in the liver, decreases plasma triglyceride concentrations, and decreases de‐novo lipogenesis in the liver when fasting.	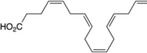	[134]
Zeaxanthin	*Chlorella*	Anti‐inflammatory	Significantly reduces disease activity index, the wet weight of the colon, the ulcer area, macroscopic grades and histological changes, effectively lowers myeloperoxidase and malondialdehyde levels, increases enzymatic activity of superoxide dismutase and catalase, increases glutathione levels, suppresses TNF‐*α*, IFN‐*γ*, IL‐6, IL‐1*β*, and NF‐*κ*B levels, digestion of nitric oxide and COX2 protein expression	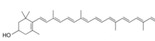	[135]
Violaxanthin	*Dunaliella tertiolecta*	Anti‐inflammatory	N/A	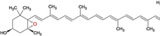	[118]
Docosapentaenoic acid (DPA)	*N. oculata*	Anti‐inflammatory	Reduces colon shortening and myeloperoxidase accumulation in the colon, inhibits abnormal production and mRNA expression of pro‐inflammatory cytokines such as TNF‐*α*, IL‐1*β*, and IL‐6, improves the production and expression of anti‐inflammatory cytokines such as IL‐10, inhibits the synthesis of leukotriene B4 (LTB4) and prostaglandin E2 (PGE2)	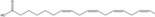	[136]

Sterols are lipids that contribute to the microalgal cell membrane's fluidity and permeability. They are used in pharmaceutical and nutraceutical products to lower blood cholesterol, with a reported 10% reduction in total cholesterol and up to 15% reduction in low‐density lipoprotein cholesterol. Cancer and inflammation are also affected positively by microalgal sterols.^[^ ^68^ ^]^ As indicated in Table 2, microalgae produce long‐chain polyunsaturated fatty acids (PUFAs) such as *γ*‐linolenic acid, arachidonic acid, eicosapentaenoic acid (EPA), and docosahexaenoic acid (DHA). For example, the food and drug administrations of the United States, China, and other countries approved *Schizochytrium* sp. as a food resource to produce DHA. *Schizochytrium* has been shown to contribute to intestinal repair through a DHA mechanism. PUFAs are found in greater concentrations in microalgae than in fish oils,^[^ ^119^, ^120^ ^]^ and the clinical use of fish oil is limited by the unpleasant fishy taste and potential contamination with heavy metals. In microalgae, n‐3 fatty acid‐derived oxidative PUFAs are produced from linoleic and alpha‐linoleic acids; their anti‐inflammatory activity consists of decreasing inflammatory cytokine levels^[^ ^121^ ^]^ and as a result, microalgae constitute a good alternative source of PUFAs.^[^ ^122^, ^123^ ^]^

Remarkable biological effects, such as anti‐inflammatory and immunosuppressive effects, were observed in glycoglycerolipids from microalgae.^[^ ^124^ ^]^ Studies on the effects of microalgal glycolipids on inflammation and cancer demonstrated their chemotherapeutic potential.^[^ ^125^ ^]^ Marine polar lipids such as glycolipids, which hold a high concentration of omega‐3 PUFAs, have been proposed as beneficial to human health. These polar lipids have higher bioavailability and are more stable than other lipids.^[^ ^126^ ^]^ Despite the limited number of microalgal species that have been approved for consumption as food, microalgal lipids are used as food ingredients, with the potential to enrich nutrient‐deficient diets.^[^ ^127^ ^]^

## 3 Effects of Microalgae‐Based Proteins and Other Bioactive Compounds on Inflammatory Bowel Disease

### 3.1 Inflammatory Bowel Disease

**Figure 2:**
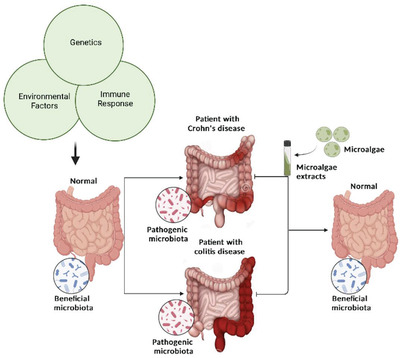
Types of IBD. Crohn's disease may develop in any area of the gastrointestinal tract, from mouth to anus, and can involve all layers of the intestine. In most patients, the inflammation is located in the final part of the small intestine. Ulcerative colitis can develop in all layers of the intestinal wall, and may involve the entire colon or only part of it, but will always include the rectum. In IBD, pathogenic microbiota is increased and beneficial microbiota are decreased. Using materials from microalgae could stop the chronic inflammatory process in the intestine and cause an increase in beneficial bacteria, thus restoring the intestine's normal state (created with BioRender.com).

IBD is a chronic inflammatory disease of the gastrointestinal tract resulting from a complex interaction of genetic, immunological, and environmental factors. Two primary forms of IBD are described: Crohn's disease and ulcerative colitis^[^ ^137^ ^]^ (**Figure** **2**). Flexibility and polarization of immune system cells through interactions with gut bacteria, metabolites, and nutritional components determine the severity of inflammation, its chronicity, and its resolution with specific targeted treatments.^[^ ^138^ ^]^ Crohn's disease can affect any part of the gastrointestinal tract, from mouth to anus. It is an idiopathic, chronic, transmural inflammatory process of the bowel. In most patients, the small bowel is involved, particularly the terminal ileum.^[^ ^139^ ^]^ Ulcerative colitis is a relapsing–remitting disease that causes inflammation and ulcers (sores) in the digestive system, affecting the innermost lining of the colon and rectum. It can involve the entire colon or just part of it, but it will always include the rectum.^[^ ^140^ ^]^ Evidence suggests that gut microbiota has a crucial role in triggering IBD.^[^ ^21^, ^141^ ^]^ Among individuals with Crohn's disease and ulcerative colitis, an imbalance has been reported in the composition of the intestinal bacteria. Normally, the beneficial intestinal microbiota produces short‐chain fatty acids (SCFAs) and vitamins, and degrades bile acids—crucial processes in mucosal barrier immunity. In IBD, these processes decrease, resulting in impaired mucosal barrier integrity, abnormal immune reactions, and intestinal inflammation.^[^ ^138^, ^142^ ^]^

Symptoms of IBD include pain in the abdomen, weight loss, diarrhea or constipation, fatigue, malnutrition, nausea and vomiting, and delayed or impaired growth in children. Symptom severity differs among individuals.^[^ ^143^ ^]^ No cure for Crohn's disease or ulcerative colitis has yet been found, but the treatment involves the use of anti‐inflammatory drugs such as 5‐aminosalicylic acid, and immunomodulators such as azathioprine, mercaptopurine, methotrexate, infliximab, adalimumab, certolizumab, and natalizumab. The existing treatments are intended to reduce the inflammatory process, regulate the immune system, relieve symptoms, and improve quality of life.^[^ ^144^ ^]^

### 3.2 Protective Effects of Microalgal Proteins, Peptides, and Bioactive Compounds against Inflammatory Bowel Disease

In the last few decades, peptides have garnered attention for their characteristic features of multifunctionality, high sensitivity, and stability. Despite the many reports on anti‐inflammatory peptides from food and food proteins, such as fish sauce,^[^ ^145^ ^]^ soy protein,^[^ ^146^ ^]^ milk protein, and beef protein,^[^ ^147^ ^]^ little has been published on microalgal proteins as a source of anti‐inflammatory peptides. Nevertheless, these latter peptides are potentially useful adjuncts in the treatment of gastric inflammation,^[^ ^59^ ^]^ and microalgae should be investigated for the industrial production of functional peptides.

The demand for new therapeutic compounds has increased research into marine invertebrates and microbes, leading to the discovery of several new compounds. The diverse population in the marine environment offers an enormous source of microbes, including bacteria, cyanobacteria, fungi, algae, microalgae, and small invertebrates.^[^ ^148^ ^]^ Traditionally, therapeutic compounds are obtained from inland organisms; however, the need for improved pharmacological compounds in recent years has led to many studies of the marine environment—including both animals and phototrophic organisms such as microalgae.^[^ ^149^ ^]^ Fatty acids, carotenoids, proteins, polysaccharides, and phenolic compounds’ antioxidant, anti‐inflammatory and anticarcinogenic properties have attracted the interest of the pharmaceutical industry, as very promising tools for the prevention of inflammatory diseases.

Wang et al.^[^ ^150^ ^]^ investigated the potential anti‐inflammatory properties of bioengineered meal hydrolysate from the marine microalgae *Schizochytrium* sp. in a murine colitis model. To determine whether the meal hydrolysate alters the expression of pro‐ and anti‐inflammatory cytokine genes, they measured the mRNA levels of interleukins (IL) 6, 10, 1*β*, and 17 and TNF‐*α*, commonly used as indicators of colitis severity. Cellular inflammation was lower in the colons of meal hydrolysate‐treated vs. control mice. The meal hydrolysate group had significantly higher levels of anti‐inflammatory IL‐10 protein expression, slightly decreased pro‐inflammatory TNF‐*α* protein expression, and significantly reduced TNF‐*α* gene expression compared to the control group, suggesting that the meal‐hydrolysate treatment inhibited acute inflammation and enhanced colon recovery from colitis.^[^ ^150^ ^]^ Microalgal compounds with therapeutic potential for IBD include tocopherols from *Porphydium* sp. and *Spirulina platensis*,^[^ ^151^ ^]^ and phenolic compounds from *Spirulina maxima*, *C. ellipsoidea*, and *Nannochloropsis* sp.^[^ ^152^ ^]^

*Spirulina platensis* phycobiliproteins demonstrate significant antioxidant and anti‐inflammatory activities.^[^ ^153^ ^]^ Moreover, microalgal lipids have garnered significant attention as biomolecules for the treatment of inflammatory pathologies such as IBD.^[^ ^154^ ^]^ Many of these are saturated or unsaturated long‐chain fatty acids, with PUFAs having been most studied for their pharmacological potential.

Phycocyanin is a typical compound found in microalgae with anti‐inflammatory and antioxidant activity. In a study evaluating the therapeutic effects and mechanisms of action of phycocyanin and phycocyanobilin in a murine colitis model, it was found that phycocyanin and phycocyanobilin have high anti‐inflammatory efficacy, even compared to the group that received mesalazine.^[^ ^155^ ^]^

*β*‐Carotene, which belongs to the carotenoid family, is provitamin A, which plays a central role in the regulation of physiological functions in the living body and is synthesized by a large variety of microalgae.^[^ ^156^ ^]^ The positive and anti‐inflammatory effects of *β*‐carotene have been observed in many studies both in vitro and in vivo,^[^ ^157^ ^]^ and these effects seem to be applicable as a treatment for IBD. Although it was reported that *β*‐carotene does not affect inflammatory markers, it was able to affect the proteomic response in Caco‐2 intestinal epithelial cells and may act to prevent diseases such as IBD.^[^ ^158^ ^]^ Recently, *β*‐carotene treatment was shown to improve the severity of colitis symptoms in a dextran sulfate sodium mouse model.^[^ ^159^ ^]^ The effect was linked to the characteristics of different pathways, such as NF‐*κ*B and COX‐2, among others, and it included a decrease in both systemic and local damage. In a study in which *Dunaliella bardawil* was fed to rats as a prophylactic, the algal extract appeared to protect against inflammation caused by acetic acid.^[^ ^160^ ^]^ There is further evidence that many IBD patients have low serum *β*‐carotene levels.^[^ ^161^ ^]^ Thus, it seems that IBD patients can benefit from consuming natural *β*‐carotene from microalgae such as *D. bardawil*.

The ketocarotenoid astaxanthin is a red pigment found in many algal^[^ ^162^ ^]^ species, such as *H. pluvialis*, *Chlorella zofigiensis*, and *Chlorococcum* sp.^[^ ^117^, ^162^, ^163^ ^]^ Astaxanthin has been used to prevent inflammatory processes such as colitis in mammalian models. According to a recent study, astaxanthin reduces inflammation by changing macrophage activity. LPS‐induced RAW 264.7 cells were used to assess its anti‐inflammatory efficacy in vitro. Before LPS treatment, cells that had been pretreated with astaxanthin exhibited a significant reduction in nitric oxide levels.^[^ ^163^ ^]^ It is worth mentioning that colon and rectal cancer are the main malignancies in IBD patients, causing 10–15% mortality in these patients. Although colon cancer patients with IBD make up only about 2% of the population with this disease, it is one of the three main risk factors for the disease.^[^ ^163^ ^]^

Lutein is a yellow oxycarotenoid found in green microalgae that protects cells from reactive oxygen species damage under stressful conditions.^[^ ^164^ ^]^ Extracts from *C. vulgaris*, whose main component is lutein, showed antiproliferative activity in human colon cancer cells (HCT‐116).^[^ ^165^ ^]^

Oxylipins are lipid mediators involved in the resolution of many inflammatory disorders. A study investigated the effects of biomass of the microalgae *Chlamydomonas debaryana* containing oxylipin on acute colitis induced by 2,4,6‐trinitrobenzenesulfonic acid in rats. The treatment with the anchored microalgae prevented colon shortening and loss of body weight reduced peripheral damage in the colon and prevented increased production of the intestinal mucosa.^[^ ^166^ ^]^ Those findings indicated that the microalgae *C. debaryana* or oxylipins derived from it can be used as nutrients in the treatment of the active phase of IBD.

## 4 The Gut–Liver Axis

### 4.1 The Gut Microbiome

Aside from its involvement in digestion and absorption, the gastrointestinal tract has immunological functions, raising defenses against infection.^[^ ^166^ ^]^ The body is constantly exposed to damage caused by viruses, parasites, bacteria, chemicals, and ingested toxins, including food supplements, medicines, and various foods. Like the skin and the respiratory system, the digestive system serves as a sterile interface between the internal environment of the body and its external environment.^[^ ^167^ ^]^ The body's defense is mostly carried out by the lymphatic system and its component lymphocyte cells. A large part of the digestive system consists of lymphoid tissue containing lymphocytes and plasma cells; the interaction between these cells residing in the tissue and the threatening factor is the basis of defense in the gut.^[^ ^168^, ^169^ ^]^

The intestinal immune system is characterized by several unique cell populations: antigen‐presenting cells and lymphocytes. Furthermore, these cells—whether epithelial cells or lymphocytes—which do not exist in other systems of the body, also show internal anatomical and functional differentiation.^[^ ^169^ ^]^ Thus, for example, inside the epithelial cells, M cells, a subset of mucosal epithelial cells, are different from the surrounding epithelial cells, and the lymphocytes inside the epithelium (intraepithelial lymphocytes) are completely different from the lymphocytes in the lamina propria which are a few microns away from them.^[^ ^170^ ^]^

As part of the functions of the gut, the gut epithelium blocks the passage of potentially deleterious undigested food substances to the underlying tissues. Intestinal protection from pathogens normally depends on non‐specific mechanisms and specific immunological responses, relying on a barrier that includes luminal, epithelial, and submucosal factors.^[^ ^171^, ^172^ ^]^ The gastrointestinal tract is one of the largest immune organs in the body; the gut‐associated lymphoid tissue contains 40% of the body's immune effector cells, and potentially 25% of the intestinal mucosal mass.^[^ ^171^ ^]^ The gastrointestinal tract has both a direct immune function with its own resident microbial flora and an immune function in distant mucosal sites, such as the liver.^[^ ^173^ ^]^ Many studies have demonstrated cross‐talk between the gastrointestinal microbiome and its host's organs, affecting local and systemic metabolism, and immune homeostasis.^[^ ^174^, ^175^ ^]^

The human gut microbiota includes bacteria, viruses, protozoa, and fungi; bacteria are the most abundant, with over 1000 species at a density of 10^11^–10^12^ cells mL^−1^. These bacteria play a fundamental role in several aspects of host homeostasis: nutrition, immunity, metabolism, and defense against pathogens.^[^ ^176^ ^]^ The bacteria *Firmicutes* and *Bacteroides* are dominant in the gut flora of a healthy host.^[^ ^177^ ^]^ The gut microbiota decomposes carbohydrates and indigestible oligosaccharides in food synthesize SCFAs such as butyric acid, propionic acid, and acetate, and provides energy for the intestinal epithelium.^[^ ^178^ ^]^

The intestinal microbiota drives the development of the gut immune system; it can induce immune homeostasis but it also contributes to the development of IBD, its dysregulation having been associated with IBD activity.^[^ ^179^ ^]^ For example, the levels of *Bifidobacterium longum* in ulcerative colitis, *Eubacterium rectale*, *Faecalibacterium prausnitzii*, *Roseburia intestinalis*, and other beneficial bacteria in Crohn's disease and ulcerative colitis are significantly reduced, whereas the relative abundance and growth rate of harmful bacteria such as *Bacteroides fragilis* is increased. *Ruminococcus torques* and other *Ruminococcus* species are also enriched in those diseases.^[^ ^180^ ^]^

Ma et al.^[^ ^181^ ^]^ tested the effects of nutritional supplements from the microalgae *Schizochytrium* sp., *Spirulina platensis*, *Chlorella sorokiniana*, *Chromochloris zofingiensis*, and *D. salina* on growth performance, immune status, and intestinal health of zebrafish (*Danio rerio*). The microalgal diet successfully regulated the intestinal microbiota of the fish and increased the relative abundance of probiotics, in addition to reducing the expression of pro‐inflammatory cytokines such as IL‐6, IL‐8, and IL‐1*β* in a normal physiological state of the intestine.^[^ ^181^ ^]^

NAFLD and IBD are frequently comorbid conditions. Both intestinal inflammation and metabolic factors are thought to contribute to NAFLD pathogenesis, especially when fibrosis and progression toward more advanced disease stages, such as non‐alcoholic hepatic steatosis, are present.^[^ ^182^ ^]^ NAFLD is characterized by an increase in *Escherichia coli* and a decrease in *F. prausnitzii*.^[^ ^183^ ^]^ It is associated with increased intestinal movement and is related to bacterial growth in the small intestine, which may link it to IBD.^[^ ^184^ ^]^

The interactions between the gut, its contents, and the liver are referred to as the “gut–liver axis,” which involves signals generated by genes, diet, and environmental factors.^[^ ^185^ ^]^ Thus, liver function, the intestine, and the immune system are intricately linked and affected by nutrients and their route of delivery.^[^ ^183^ ^]^ The gut–liver axis seems to play a significant role in the body's response to sepsis, systemic inflammation, and multiple organ dysfunction.

In addition, unusual processes and unique cells and molecules combine to allow the intestinal immune system to function and respond selectively to the enormous number of antigenic stimuli to which it is exposed.^[^ ^186^ ^]^ Harmful antigens and pathogens are detected and eliminated, while beneficial or harmless antigens and microorganisms are identified and oral tolerance to them develops. The same cellular mechanisms and molecules—oral tolerance, controlled inflammation, the physical barrier made up of the epithelial cells and mucin layer, and more, are responsible for this miraculous functioning of the intestinal immune system.^[^ ^187^ ^]^ Dysfunction of one or more of the components of the intestinal immune system may result in significant pathologies, such as food intolerance and IBD.^[^ ^188^ ^]^

### 4.2 The Gut–Liver Axis

**Figure 3:**
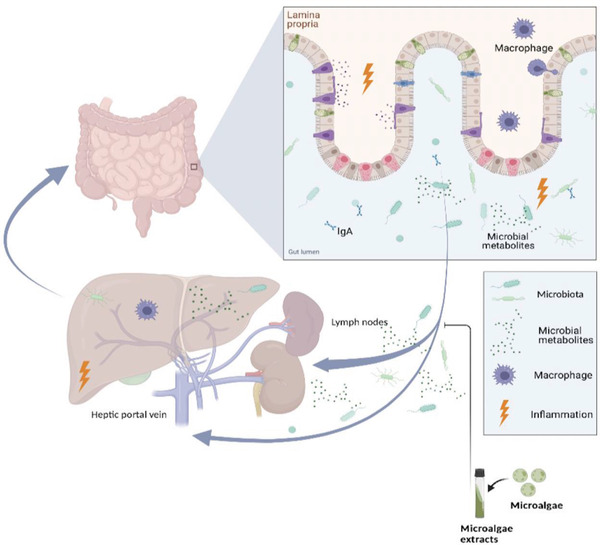
Gut–liver axis pathogenesis. Gut microbiome composition is influenced by environmental factors (including diet), genetic factors, and the mucosal immune system. In dysbiosis, increased intestinal permeability, chronic systemic inflammation, production of SCFAs, and changes in metabolism, all caused by the intestinal microbiota and their products, may contribute to the development of liver diseases. In the dysbiotic intestinal environment, microbial metabolites and host factors hold equal importance in the pathogenesis of liver disease (created with BioRender.com).

Through the gut–liver axis, changes in gut bacteriology and physiology can affect the hepatocellular function and systemic immunity (**Figure** **3**).^[^ ^189^, ^190^ ^]^ Several processes govern the interactions between the gastrointestinal tract and the liver, and peptides from the gut seem to be involved in liver regeneration. Factors driven from the intestine after extensive bowel resection or functional exclusion of the small intestine may lead to severe liver dysfunction and even cirrhosis.^[^ ^190^ ^]^ The liver appears to have metabolic interactions with the stomach, the colon, and the intestine.^[^ ^191^, ^192^ ^]^

The liver is an essential organ in the metabolism of cytokines, which are involved in the liver's physiological functions and homeostasis^[^ ^193^ ^]^ and are elevated in liver disease, contributing to multiorgan failure.^[^ ^194^ ^]^ Cytokines produced by liver cells modulate most of this organ's metabolism of amino acids, proteins, lipids, carbohydrates, and trace minerals.^[^ ^195^ ^]^ They mediate anabolic and catabolic functions, and regulate hepatic blood flow, bile flow, liver regeneration and the response to ischemia‐reperfusion injury. Because nutrition can influence cytokines and prostanoid release by Kupffer cells,^[^ ^196^ ^]^ it also affects hepatocellular function and response to injury and infection.^[^ ^197^ ^]^ Endotoxin‐stimulated monocytes and macrophages produce inflammatory mediators and cytokines, and some of these affect DNA synthesis. Acute liver failure is commonly accompanied by bacterial infection,^[^ ^144^ ^]^ with increases in the production and release of cytokines such as TNF‐*α*. There is significant inhibition of DNA synthesis in the liver, with a marked increase in IL‐1*β* and serum IL‐6 concentrations.^[^ ^198^ ^]^

The gut bacteria are key to maintaining Kupffer cell responses, and the overgrowth of Gram‐negative bacteria enhances their response to LPS. Upon bacterial overgrowth in the intestine, intestinal atrophy, and increased gut permeability, bacteria, and endotoxins are thought to move from the intestinal lumen to the mesenteric lymph nodes and portal circulation; there, they stimulate the release of inflammatory mediators by peritoneal, intestinal, and hepatic macrophages.^[^ ^199^ ^]^

### 4.3 The Protective Effect of Microalgal Proteins, Peptides, and Bioactive Compounds on the Gut Microbiome and the Gut–Liver Axis

As gut health and gut microflora is essential to human health and provide protection against diseases such as IBD, the potential benefits of microalgae in restoring the dysfunction of the gut microbiome in the case of gut inflammation are being explored.^[^ ^185^ ^]^

Probiotic is a general term for organisms living in food and nutritional supplements (mostly bacteria) that are consumed by humans to improve health beyond the consumption of basic nutrients. These bacteria are safe for consumption and can survive bile and stomach acids.^[^ ^200^ ^]^ In addition, they regulate the immune system, contribute to the normal functioning of the epithelial barriers and also have a positive effect on the intestinal flora.^[^ ^201^ ^]^ Prebiotics is the term used for carbohydrate compounds consumed as food that are indigestible, such as germinated barley, lactosucrose, psyllium, and more.^[^ ^202^ ^]^ The role of these compounds is to improve metabolism and encourage the growth of protective endogenous bacteria. In addition, these compounds are involved in changes in SCFAs of the large intestine by fermentation of its bacterial population.^[^ ^203^ ^]^

Probiotics can affect the gut microbiome by providing some of the beneficial immunomodulatory effects of the commensal gut microbiota and inducing immune homeostasis. Probiotic administration has been proposed as a suitable treatment for mild to moderate IBD. Probiotics as IBD prophylactics are conceived based on the role of intestinal bacteria in the pathogenesis of the disease.^[^ ^204^ ^]^ The most common probiotic bacteria are *lactobacilli*, *lactococci*, *bifidobacteria*, *enterococci*, and *streptococci*, as well as yeasts such as *Saccharomyces bouldarii*. There are recent indications of newly identified health‐promoting bacteria being associated with positive effects on their host. One example is *F. prausnitzii*, the supplementation of which is inversely correlated with the incidence of IBD, and in particular Crohn's disease.^[^ ^205^ ^]^ The understanding that there is a close relationship between the development of IBD and the microflora in the intestines has led to many studies that have tested the therapeutic potential of probiotics and/or prebiotics.^[^ ^206^ ^]^

Oligosaccharides derived from algae can be used as prebiotics to prevent IBD.^[^ ^207^ ^]^ For example, in a study of algal extract (laminarin and fucoidan) supplementation to pigs, the extracts appeared to reduce *Salmonella typhimurium* levels compared to galactooligosaccharide, a prebiotic derived from lactose in cow's milk, which did not reduce the growth of pathogenic microorganisms.^[^ ^208^ ^]^ Moreover, polysaccharides and polyphenols, which are also found in microalgae, have a beneficial effect on gastrointestinal strains of *Bifidobacterium* and *Lactobacillus*, which are known to confer a health benefit to the host.^[^ ^209^ ^]^ Ingestion of polysaccharides, oligosaccharides, and dietary fibers can help maintain a healthy intestinal microbiome.^[^ ^210^ ^]^

There is a growing interest in exploring microalgae's prebiotic effects on gut health due to their higher fiber and carbohydrate contents and additional phytochemicals, particularly oligosaccharides.^[^ ^211^ ^]^ Agal‐based prebiotics may combat IBD due to their antagonistic activity against pathogenic microbes and their efficacy in stimulating the growth of beneficial organisms and bestowing an anti‐inflammatory status to the immune cells.^[^ ^212^ ^]^ An example of this is polysaccharides taken from *Himathalia elongata* that produced propionate in vitro, which were used in the liver as a cholesterol‐lowering compound.^[^ ^213^ ^]^ Bhowmik et al. showed the prebiotic effect of *Spirulina platensis* on lactic acid bacteria as it increased their growth while having potent antibacterial activity against human pathogenic bacteria; this illustrated its potential to balance the gut flora. *A. platensis* and *C. vulgaris* also significantly increased the viability of *Lactobacillus acidophilus* and *bifidobacteria*.^[^ ^214^ ^]^

Recent studies have shown that a high‐fat diet may lead to an imbalance in the gut microbiome. Yu et al, showed that *Spirulina platensis* can significantly reduce the relative amount of Firmicutes/Bacteroidetes in rat fecal samples, in addition to regulating intestinal permeability, resulting in a significant reduction in intestinal inflammation in rats fed a high‐fat diet.^[^ ^215^ ^]^ Another study evaluated the stimulatory effect of *S. platensis* biomass on the growth of *L. acidophilus*. In addition to its metabolic activity during fermentation for 72 h at 37 °C, the effect of *S. platensis* biomass was more widespread than that of the accepted prebiotic fructooligosaccharide. Its supplementation resulted in the proliferation of *L. acidophilus* and an increase in metabolic activity.^[^ ^216^ ^]^

Other algal phytochemicals, including fatty acids, pigments, and polyphenols, and prebiotic features of polysaccharides could serve as interventional dietary strategies to combat non‐communicable diseases.^[^ ^217^ ^]^ Studies have shown that oral intake of polyphenols stimulates the growth of prebiotics, which then leads to a decrease in fat percentage, body weight, and liver steatosis, and a positive effect on glucose metabolism.^[^ ^218^ ^]^

In dysbiosis, damaging bacteria grow to higher levels than healthy bacteria, and factors such as LPS contribute to the progression of gut inflammation and its effect on the liver.^[^ ^219^ ^]^ The glyceroglycolipids monogalactosyldiacylglycerol and digalactosyldiacylglycerol, extracted from *T. chuii* and *Nannochloropsis* sp. *granulata*, successfully reduced nitric oxide activity in LPS‐stimulated models of RAW 264.7 and THP‐1 macrophages, while *Isochrysis galbana* extract lowered TNF‐*α* production.^[^ ^220^, ^221^ ^]^ Two methanol fractions from *Cylindrotheca closterium* were recently shown to have TNF‐*α*‐suppressive activity in an LPS‐stimulated human monocyte THP‐1 cell model. The anti‐inflammatory activity was attributed to a choline‐containing phospholipid known as lysophosphatidylcholine and the chlorophyll‐related hydroxypheophorbide *a*.^[^ ^222^ ^]^

Tzachor et al. observed a *Spirulina* extract's inhibitory effect on LPS‐induced TNF‐*α* in both macrophages and monocyte cells, and attributed it to a possible synergistic effect between functional groups that might include C‐phycocyanin, sorbitol, and adenosine derivatives.^[^ ^41^ ^]^

## 5 Non‐Alcoholic Fatty Liver Disease and the Effect of Microalga‐Based Proteins and Bioactive Compounds

### 5.1 Non‐Alcoholic Fatty Liver Disease

**Figure 4:**
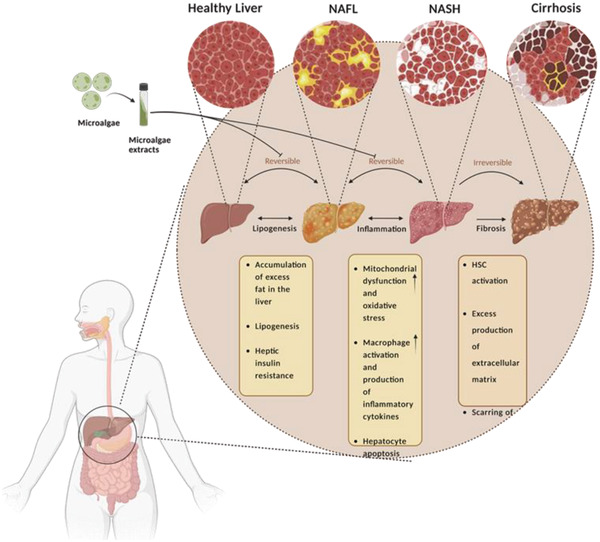
Stages of NAFLD development and progression. NAFLD refers to a range of liver conditions that affect people who drink little to no alcohol. There are three stages of NAFLD development: Simple steatosis (or NAFL), NASH, and liver cirrhosis. Hepatic steatosis is brought on by the accumulation of excess fat in the liver, lipogenesis, and systemic insulin resistance. Mitochondrial dysfunction and oxidative stress, inflammation, and hepatocyte apoptosis contribute to the development of NASH. Liver fibrosis in NASH is a transitional phase that results in the development of liver cirrhosis (created with BioRender.com).

NAFLD is considered to be the hepatic manifestation of metabolic syndrome; it is tightly associated with metabolic co‐occurrences, such as obesity, type 2 diabetes, hyperlipidemia, and hypertension. In fact, the main cause of chronic liver disease is metabolic syndrome, estimated to affect ≈25% of the adult population worldwide.^[^ ^223^ ^]^ NAFLD is a progressive disease. The first stage consists of the accumulation of excess lipids in the hepatocytes. It can then evolve to a more complex stage termed non‐alcoholic steatohepatitis (NASH), consisting of hepatic steatosis, hepatocellular damage, inflammation, and varying degrees of fibrosis (**Figure** **4**).^[^ ^224^ ^]^ The clinical manifestations and evolution of NASH are quite heterogeneous.^[^ ^225^ ^]^ Cases of NASH with advanced fibrosis (occurring in 10–20% of NAFLD cases)^[^ ^226^ ^]^ may further progress to cirrhosis, hepatocellular carcinoma (HCC), and end‐stage liver disease, and these individuals could require a liver transplant. NASH is therefore a critical step in the clinical progression of NAFLD.^[^ ^227^ ^]^ A key challenge in this disease's management is the identification of high‐risk patients; this can also serve to improve patient selection in the design of clinical trials for emerging pharmacotherapies. Globally, a major health challenge consists of decreasing NASH onset and preventing its transition to liver failure and HCC.^[^ ^228^ ^]^

The etiology of NASH, a complex multifactorial disease, is not understood. The hallmarks of NASH—steatosis, liver injury, and inflammation—which lead to the onset of fibrosis and sometimes HCC, have several triggers (Figure 4).^[^ ^229^ ^]^ Steatosis frequently occurs early in NAFLD, but other stresses are needed to induce NASH onset, including lipotoxicity, oxidative stress, and inflammation; together, these induce cellular stress pathways, resulting in hepatocyte death, inflammation, and fibrosis development.^[^ ^230^ ^]^ The inflammatory environment in NASH plays a vital role, in that inflammatory mediators such as TNF‐*α*, produced by immune cells, kill the lipid‐loaded, stressed hepatocytes which have become sensitive to cytokine‐mediated cell death.^[^ ^231^ ^]^

In the lipid‐overloaded liver, fat types and the ways in which the liver cells handle these lipids may result in adaptation through the development of isolated hepatic steatosis or may trigger cell death by various distinct molecular mechanisms. In the latter situation, the hepatocytes release stress signals, also called danger signals which, in the absence of infection, activate sterile inflammatory pathways. Over time, this results in chronic injury and an abnormal wound‐healing response with fibrosis.^[^ ^223^ ^]^

During lipotoxicity, the stressed or dying hepatocytes may release damage‐associated molecular patterns (DAMPs)—intracellular molecules that act on the liver's immune cells to repair tissue injury through a homeostatic, wound‐healing response. However, if these signals persist, the result may be a full‐blown inflammatory response, with tissue inflammation and excessive scarring, leading to advanced fibrosis and ultimately, cirrhosis.^[^ ^228^ ^]^

Pathogen‐associated molecular patterns (PAMPs) also have a role in determining liver injury in NAFLD/NASH. These are bacterial products, including LPS, originating from the cell wall of Gram‐negative bacteria, as well as peptidoglycans, bacterial lipoproteins, flagellins, and bacterial RNA and DNA.^[^ ^229^ ^]^ PAMPs reach the liver through disruptions in the intestinal mucosal barrier and activate innate immune cells; this causes intracellular signaling cascades that amplify the injury, thus triggering the inflammatory response that results in organ damage, mediated mainly by the production of pro‐inflammatory cytokines such as TNF‐*α* and IL‐6, which influence insulin resistance and lipid metabolism.^[^ ^232^ ^]^ PAMPs and DAMPs are recognized by pattern recognition receptors, the best characterized of which are the Toll‐like receptors (TLRs).^[^ ^233^ ^]^ These are a family of cell surface and endocytic receptors that are expressed in most liver cells, including hepatocytes, Kupffer cells, hepatic stellate cells, biliary epithelial cells, and sinusoidal endothelial cells, with each cell population having its own specific pattern of TLR expression.^[^ ^234^ ^]^ In the liver, and especially in NASH, the best‐studied TLRs are TLR2, TLR4, and TLR9,^[^ ^235^ ^]^ recognizing peptidoglycans, bacterial LPS, and bacterial DNA, respectively.^[^ ^236^ ^]^ The role of TLRs in NAFLD/NASH has been studied using genetic ablation of these proteins in mice.^[^ ^232^ ^]^

The liver contains a large number of innate and adaptive immune cells, making it an important immunological organ, in addition to being essential for detoxification.^[^ ^233^ ^]^ The liver's high vascularization and reduced blood flow in fenestrated capillary‐like vessels maximize immune cell exposure to blood‐borne and gut‐derived pathogens.^[^ ^237^ ^]^ About one‐third of the body's blood volume flows through the liver every minute, most coming from the portal vein and draining into the gastrointestinal tract.^[^ ^238^ ^]^ The portal vein delivers intestinal immune cells, cytokines, and gut‐derived products directly to the liver.^[^ ^185^ ^]^ This interdependency of the gut and liver explains why damage to the intestinal barrier, allowing the flow of microbiota components and metabolites into the liver, leads to or exacerbates liver diseases.^[^ ^184^ ^]^

The intestinal barrier in the gut–liver axis consists of physical, immune, and biochemical components.^[^ ^195^ ^]^ A physical barrier is created by the gut's vascular cells and simple epithelium, linked by tight junction proteins, the mucus layer, and microorganisms. The biochemical barrier is made up of bile acids and antimicrobial proteins.^[^ ^182^ ^]^ The immune barrier consists mainly of secreted immunoglobulin A and lymphoid follicles containing a variety of immune cells.^[^ ^183^ ^]^ The intestinal barrier is generally the first line of defense in human immunity; the liver provides the second line of defense against pathogenic factors that pass through the intestinal barrier.^[^ ^184^ ^]^ The immune tissues in the intestine and liver participate in immune tolerance to food antigens and pathogen clearance.^[^ ^239^ ^]^ Gastrointestinal microbiome dysbiosis can disrupt these barriers through an increase in mucosal permeability. In addition to modifying the intestinal microbiome, dietary compounds can play an essential role in maintaining or damaging the intestinal barrier. Barrier damage as a consequence of a high‐fat diet can result in the translocation of intestinal bacteria and endotoxins through the portal venous system.^[^ ^240^ ^]^ These will activate the immune cells in the liver to release host inflammatory factors, which will damage tissues in the intestinal mucosa, liver, and systemic organs.^[^ ^228^ ^]^ The gastrointestinal microbiome and its metabolites have a range of effects on liver health and disease, and the promotion of liver health remains a major concern.^[^ ^240^ ^]^

### 5.2 The Protective Effect of Microalgal Proteins, Peptides, and Bioactive Compounds on Non‐Alcoholic Fatty Liver Disease

Marine‐derived bioactive peptides, including compounds derived from microalgae, have been reported to play a vital role in NAFLD. Therapeutic bioactivities—hepatoprotective, anti‐inflammatory, immunomodulatory, and anticancer—have been described for proteins from *Spirulina platensis* and *Porphyridium* sp.^[^ ^241^, ^242^, ^243^ ^]^

*C. vulgaris* powder was given as tablets to individuals with NAFLD. The supplement reduced body weight, improved blood sugar status and liver function, and reduced C‐reactive protein levels. In addition, it downregulated inflammatory genes in the p38 mitogen‐activated protein kinase (MAPK)/TNF‐*α*/NF‐ƙB pathway, supporting the use of *C. vulgaris* supplements as potential adjuvant therapy for NAFLD.^[^ ^243^ ^]^

Kumar et al. examined the effect of a *Scenedesmus dimorphus*–*Schroederiella apiculata* blend containing 46.1% protein, 19.6% insoluble fiber, and 2.8% omega‐3 fatty acids on rats fed a high‐carbohydrate and high‐fat diet. They found complete prevention of the increased liver weight, abundant enlarged fat vacuoles, and inflammatory cell infiltration observed in the control group that did not consume the microalgal blend.^[^ ^244^ ^]^ In addition, the increased values of alanine aminotransferase and aspartate aminotransferase (AST) in the control group, which indicate liver damage, were decreased to normal levels in rats treated with the microalgal blend. Finally, improved glucose tolerance and insulin sensitivity in the treatment group indicated possible prevention of steatosis development.^[^ ^245^ ^]^ A recent study showed the preventive effects of *Tisochrysis lutea* on NAFLD. Wistar rats were fed a standard diet, a high‐fat and high‐fructose diet (HF) (10% fructose in the drinking water), or the HF diet supplemented with 12% *T. lutea* extract, for 8 weeks. The *T. lutea*‐supplemented group showed a decrease in body weight, abdominal and epididymal adipose tissues, liver triglyceride content and plasma AST level compared to the other groups. The algal supplementation also lowered plasma glucose, an indication of reduced insulin resistance, and TNF‐*α* was significantly reduced, indicating the supplement's potential for the treatment of NAFLD.^[^ ^245^ ^]^

Kang et al. looked at the protective effect of *Navicula incerta* peptides Pro‐Gly‐Trp‐Asn‐Gln‐Trp‐Phe‐Leu (NIPP‐1) and Val‐Glu‐Val‐Leu‐Pro‐Pro‐Ala‐Glu‐Leu (NIPP‐2) against liver disease.^[^ ^83^ ^]^ Peptides NIPP‐1 and NIPP‐2 were purified from *N. incerta* protein hydrolysate and their inhibitory effects on collagen release were examined in hepatic stellate LX‐2 cells with transforming growth factor (TGF)‐*β*1‐activated fibrosis.^[^ ^83^ ^]^ NIPP‐1 peptide dose‐dependently prevented elevations in matrix metalloproteinase levels and inhibited TIMP metallopeptidase inhibitor 1 production. Both NIPP‐1 and NIPP‐2 prevented hepatic fibrosis by inhibiting TGF‐*β*1‐stimulated fibrogenesis, suggesting their potential as an antifibrosis treatment. Addition of these peptides clearly reduced the amount of collagen, an important indicator of hepatic fibrinolysis, showing hepatoprotective activity.^[^ ^246^ ^]^ Ma et al. investigated the effect of phycocyanin on NAFLD by evaluating the expression of peroxisome proliferator‐activated receptor (PPAR)‐alpha and sterol regulatory element‐binding protein 1, which are responsible for the regulation of lipid metabolism in the liver; they found a reduction of lipid accumulation in the liver and improvement of NAFLD.^[^ ^247^ ^]^ The phycocyanin effectively reduced the levels of SERBP‐1, which is responsible for increased unsaturated fatty acid production and its gene's transcription in the liver, and increased the levels of PPAR‐alpha, which significantly enhances the oxidation of fatty acids and inhibits the synthesis of triglycerides.^[^ ^247^ ^]^

Fucoxanthin may provide health benefits for the prevention of chronic diseases such as liver diseases. Studies have examined fucoxanthin's inhibitory effects on hepatic lipid formation. Fucoxanthin supplemented at doses of 0.05% or 0.2% on a per weight basis for 6 weeks drastically reduced hepatic triglyceride and cholesterol buildup in C57BL/6J mice fed a high‐fat diet (20% fat, wt/wt).^[^ ^248^ ^]^ This effect was probably mediated by a decrease in hepatic lipogenesis and an increase in hepatic lipolysis. Fucoxanthin also significantly suppressed activities of enzymes related to fatty acid formation and stimulated the expression of genes related to fatty acid *β*‐oxidation in the liver. As such, it could serve as a potential therapeutic compound for the treatment of NAFLD.^[^ ^249^ ^]^

Due to their valuable biological and health‐beneficial functions, marine microalga‐derived bioactive peptides thus have the potential as active ingredients in various functional foods, and nutraceutical and pharmaceutical products.^[^ ^250^ ^]^

In addition to the effects of microalga‐based proteins and peptides, fatty acids produced by the microalgae *N. oculata*, including EPA and DHA, were shown to decrease TLR4, and Robertson et al. showed inhibition of LPS‐induced inflammatory pathways in human macrophages by lipid extracts of *Pavlova lutheri*.^[^ ^251^ ^]^ Moreover, other microalga‐based active compounds—such as carotenoids, proteins, polysaccharides, triterpenoids, and phenolic compounds—may have an effect on receptors, including TLR4, TLR2, and TLR9.^[^ ^118^ ^]^ Suppression of the TLR4, which binds LPS, and of TLR9, which binds DNA derived from intestinal bacteria, markedly attenuates the inflammatory phenotype in a number of experimental models of NAFLD/NASH.^[^ ^185^ ^]^

## 6 Potential Impacts and Barriers

In recent years, microalgae have become essential crops due to their positive impact on nutrition, health, and the environment. However, the microalgal industry is facing challenges and barriers, mostly brought on by governmental legislation.

### 6.1 Environment

Rapid population growth, expected to reach 9.6 billion by the year 2050, will soon pose a barrier to fulfilling daily protein consumption requirements. Today, animal‐based products such as meat, dairy, and eggs are the main source of dietary protein, and by the year 2050, the demand for meat is expected to increase by 73%, that is, 455 million tonnes.^[^ ^252^ ^]^ To overcome this issue, the search for sustainable alternative protein sources that will supplement animal feed and food products is of high priority.^[^ ^252^ ^]^

Microalgae have been shown to have great potential as an alternative protein source given their ability to easily adapt to different ecological niches with minimal harm to the environment compared to land‐grown crops. Microalgae's rapid carbon‐dioxide fixation compared to plants has garnered a great deal of attention. It means that they can be grown on an industrial scale with low greenhouse gas emissions, and convert inorganic carbon, nitrogen, and phosphorus into biomass.^[^ ^102^ ^]^ However, despite their high diversity, only a few microalgal species are used in industrial production and are available commercially, mainly *Chlorella* spp., *Nannochloropsis* spp., *Hematococcus pluvialis*, *Spirulina* spp. and *Arthrospira* spp. Others, such as *Tetraselmis* spp., *T. lutea*, *Dunalliela salina*, *P. tricornutum*, *Porphyridum* spp., and *Scenedesmus* spp. are cultured by several companies in Europe. *Spirulina*, for example, has been successfully grown for over 50 years using several methods and equipment under a broad range of geographical conditions, including open and closed systems such as indoor tubular vertical columns, or flat‐plate photobioreactors and outdoor raceway ponds.^[^ ^106^ ^]^ Controlled environment agriculture methods have played a significant role in terms of efficiency, yield, quality traits, biomass nutritional composition, organoleptic characteristics, manufacturing costs, and the environmental impact of biomass and culture conditions.^[^ ^106^, ^253^ ^]^ For instance, only a small amount of fresh water is lost through evaporation in closed *Spirulina* photobioreactors, which impacts production inputs, cost, pathogen contamination threats, and the environment.^[^ ^254^ ^]^ Microalgae with high photosynthetic efficiency may grow in distant areas using non‐potable wastewater, and without relying on climatic conditions. One of the hindrances to commercial microalgal cultivation is the high cost of microalgal culture systems (photobioreactors), which provide the light needed by cultures typically living under autotrophic conditions.^[^ ^79^ ^]^ This can be overcome by growing microalgae heterotrophically, through the provision of an organic source to the medium. However, the maximum specific growth rate under heterotrophic conditions can be lower than under phototrophic growth.^[^ ^255^ ^]^ Another microalgal sustainability issue is harvesting and dewatering. A great deal of energy is used on dewatering due to microalgae's low solids concentration (0.6–0.9 g L^−1^) and this may be one of the main reasons for the commercialization bottleneck.^[^ ^256^ ^]^ For protein production, this issue could be solved by using wet biomass, though this would require extra pretreatment with the use of more solvents for protein extraction.^[^ ^257^ ^]^ The issue of solvent extraction, the most common method for production,^[^ ^77^ ^]^ raises problems related to energy consumption, environmental pollution, and safety risks. To increase extraction efficiency, the solvent needs to be heated, making the process even more energy‐intensive.^[^ ^79^ ^]^ This can be alternatively approached by using environmentally friendly and cost‐effective extraction methods such as the biorefinery cascade approach^[^ ^258^ ^]^ or the Supercritical CO2 extraction process.^[^ ^259^ ^]^ The aquaculture industry poses yet another environmental problem. In providing food for humans, it also generates a large amount of wastewater, threatening global sustainability.^[^ ^260^ ^]^ A large number of required antibiotics, water replacement, and expensive filters create safety problems and higher costs. To overcome these issues, the aquaculture industry is investing in the application of microalgae for wastewater remediation, biomass production, and water quality control.^[^ ^261^ ^]^

In the feed industry, the increase in meat demand will be accompanied by an increase in crop demand to feed the animals. The cow and poultry industry consumes more food than it produces and more than 75% of agricultural crops are used for animal feed.^[^ ^262^ ^]^ The crops today take up a large amount of arable land, water, and chemicals to prevent plant diseases. Using microalgae as a source of food and protein for animals can help reduce the expected land damage by providing feed ingredients as an alternative to corn and soy with the same value in a more sustainable manner.^[^ ^45^ ^]^

### 6.2 Legislation

Microalgae can be used to synthesize value‐added components such as proteins and other bioactive compounds^[^ ^260^, ^261^ ^]^ for use in the food and pharmaceutical industries, but they are still considered to be unconventional foods, and their lack of toxicity needs to be established through testing. National regulations vary among countries, leading to the publication of recommendations and toxicological evaluations by various international organizations. As part of the toxicological assessment, the algal material is mainly analyzed for compounds synthesized by the algae itself, such as nucleic acids and toxins, or compounds that are accumulated from the environment, such as heavy metals. Microalgal production for human nutrition is subject to a range of regulations. International testing programs have been established, being either required or recommended, for unconventional foodstuffs such as single‐cell proteins.^[^ ^64^ ^]^

Two of the main barriers to positioning novel microalgae‐derived proteins or peptides in the market are European legislation and the FDA. Technological, regulatory, and market‐related aspects limit production.^[^ ^262^ ^]^ Microalgae are considered a novel food, and applications need to be filled out for new species before their introduction to consumers. Prior to 15 May 1997, when the first regulation (Regulation EC No 258/97) on novel foods was issued, a novel food was characterized as a food that had not been consumed to a significant degree by humans in the European Union.^[^ ^64^ ^]^ In 2018, additional novel food legislation—Regulation (EC) 2015/2283—came into effect, referring to the approval of foods derived from ingredients or production processes that were not in use in the European Union prior to 15 May 1997.^[^ ^263^ ^]^ Alternative proteins may also be regulated through the Food, Drug, and Cosmetic Act or by their assessment for GRAS status.^[^ ^264^, ^265^ ^]^

The process for submitting and gaining approval for an ingredient as a novel food is time‐consuming (taking around 4 years) and costly (300K–400K euros), which makes the process difficult for most small‐ and medium‐sized enterprises (https://www.efsa.europa.eu/en/applications/novel‐food‐traditional‐food). This is a major barrier to the food market's use of algal ingredients^[^ ^266^ ^]^ and to date, only a very limited number of microalgal strains and ingredients have been approved as novel foods: only 6 microalgal species and 4 microalga‐based ingredients in 2021. Most new food applications for microalgae are based on *Spirulina platensis* and *Chlorella*, because a long history of consumption means that these algae do not require approval as novel foods. *T. chuii* was approved as a novel food in 2014, and approval for *Nannochloropsis* is pending.^[^ ^267^ ^]^

### 6.3 Market Potential

According to a new report published by Allied Market Research on the algal product market (https://www.alliedmarketresearch.com/press‐release/algae‐products‐market),^[^ ^268^ ^]^ the global algal products market is expected to generate 4,286.8 million USD by 2031, with a compound annual growth rate (CAGR) of 4.88%. The increasing demand for algae is supported by a rise in the global population, increasing consumer awareness of healthy food products, and protein shortage. The global algal protein market is expected to reach 6.46 billion USD with 8.4% CAGR by the year 2030 (https://www.grandviewresearch.com/press‐release/global‐algae‐protein‐market).^[^ ^269^ ^]^

Important considerations before investing in an algal product business include: i) The potential market for the algal product, ii) possible competition with non‐algal sources, iii) the time and cost of obtaining approval for the new product, and importantly, iv) its acceptance by consumers.^[^ ^270^ ^]^ Market prices for algal biomass and its valued components fluctuate strongly at the global production level, in the actual marketplace, and especially as regards production purity.^[^ ^271^ ^]^

Due to *Spirulina* and *Chlorella*’s GRAS status, high vitamin, mineral, and carotenoid contents, and significant benefits as sources of protein‐rich and functional foods, these microalgae are sold directly as dietary supplements, reaching sales of 30–70 USD kg^−1^ for *Spirulina* and 25–50 USD kg^−1^ for *Chlorella* in 2021.^[^ ^262^ ^]^ In 2019, the United States led the world in algal production, followed by Canada and Mexico.

IBD and NAFLD create an enormous economic and medical burden due to high direct healthcare costs and indirect costs, for example, sick leave and work disability. In the United States, patients with IBD are expected to incur total lifetime costs of ≈900 billion USD.^[^ ^272^ ^]^ It is estimated that over 64 million people have NAFLD, with annual direct medical costs of about 103 billion USD and another 188 billion USD in related societal costs.^[^ ^273^ ^]^ In Europe, the economic burden and permanent work disability for IBD are high, with an annual direct healthcare cost of 4.6–5.6 billion euros. Unemployment (10%), sick leave (3–6 weeks per year), and permanent work disability (twice as high as in the healthy population) are more common in patients with IBD than in unaffected individuals.^[^ ^274^ ^]^ There are over 52 million people with NAFLD in Germany, France, Italy, and the United Kingdom, with an annual cost of about 35 billion euros.^[^ ^275^ ^]^ Introducing microalgae into the IBD and NAFLD markets would open up new economic avenues that could potentially reduce IBD‐ and NAFLD‐related healthcare costs and clinical burden. There is therefore a growing interest in alternative solutions, including functional food nutraceuticals.^[^ ^276^ ^]^ Due to the absence of any effective treatments, funding for NAFLD and IBD research reflects the critical need to find alternative solutions; microalgae may offer a novel approach.^[^ ^277^ ^]^

Another point to be considered is the development of efficient biomass‐production strategies and high‐value bioactive materials for unicellular organisms such as microalgae. It turns out that under different carbon/nitrogen conditions, mixotrophy combined with ventilation in a column photobioreactor can improve the biomass yield of certain microalgae, as shown by Shaohua et al. for *P. purpureum*.^[^ ^278^ ^]^ A high carbon‐to‐nitrogen ratio (HC/N) can promote the degradation of phycobiliprotein present in cyanobacteria and certain microalgae during nitrogen limitation; this procedure provides a sufficient amount of nitrogen to maintain anabolic metabolism.^[^ ^278^ ^]^ As a result, desired fats and polysaccharides accumulate and high excretion of polysaccharides and efficient synthesis of fatty acids such as *ω*‐6 PUFAs are obtained. Increasing the carbon‐to‐nitrogen ratio through the addition of glycerol under nitrogen‐limiting conditions may address the difficulties in obtaining low biomass and increase the desirable yield. It is worth noting that further studies are needed, with other microalgae as well, for a better understanding of the molecular mechanisms of this effective regulation method.^[^ ^278^ ^]^

## 7 Conclusion

The western lifestyle, growing population, and consumption of animal‐based products have introduced new diseases and challenges. In addition, the COVID‐19 pandemic and the current war in the European Union, which are affecting food security globally, highlight the need for the food industry to find alternative solutions for protein production.

To benefit a sustainable and healthy human life, researchers are investing efforts in finding novel food ingredients and protein sources to combat diet‐based disease. IBD and NAFLD are two chronic inflammation‐based disorders that have a significant impact on the global population.

The economic burden, the impact on the quality of patient's lives, and the adverse effects of the current inflammation‐alleviation strategies have prompted the need to find alternative dietary interventions. Microalga‐related proteins, peptides, and bioactive compounds have the potential to be used as sustainable sources of proteins to substitute animal‐based products and to prevent and decrease the prevalence and burden of food habit‐related disorders such as IBD and NAFLD.

## Conflict of Interest

The authors declare no conflicts of interest.
